# Supplementary material for: Miniaturized structured illumination microscopy using two 3-axis MEMS micromirrors
Source: Biomed Opt Express. 2022 Nov 15;13(12):6443–56. doi: 10.1364/BOE.475811 (PMC9774859; doi:10.1364/BOE.475811)
Supplement: Supplementary file 1 [file boe-13-12-6443-s001.pdf]

Supplemental Document

Biomedical Optics EXPRESS

Miniaturized structured illumination microscopy using two 3-axis MEMS micromirrors: supplement

PETER TINNING,[1,2] MARK DONNACHIE,[1] JAY CHRISTOPHER,[1] DEEPAK UTTAMCHANDANI,[1] AND RALF BAUER[1,*]

[1] *Centre for Microsystems and Photonics, Department of Electronic and Electrical Engineering, University of Strathclyde, 99 George Street, Glasgow, G1 1RD, UK*
[2] *Currently with the Department of Physics, University of Strathclyde, 107 Rotten Row, Glasgow, G1 1XJ, UK*
[*] *ralf.bauer@strath.ac.uk*

This supplement published with Optica Publishing Group on 15 November 2022 by The Authors under the terms of the Creative Commons Attribution 4.0 License in the format provided by the authors and unedited. Further distribution of this work must maintain attribution to the author(s) and the published article's title, journal citation, and DOI.

Supplement DOI: https://doi.org/10.6084/m9.figshare.21518046

Parent Article DOI: https://doi.org/10.1364/BOE.475811

Miniaturized structured illumination microscopy using two 3-axis MEMS micromirrors: supplemental document

PETER TINNING,[1,2] MARK DONNACHIE,[1] JAY CHRISTOPHER,[1] DEEPAK UTTAMCHANDANI,[1] AND RALF BAUER[1,*]

[1]*Centre for Microsystems and Photonics, Department of Electronic and Electrical Engineering, University of Strathclyde, 99 George Street, Glasgow, G1 1RD, U.K.*
[2]*Currently with the Department of Physics, University of Strathclyde, 107 Rotten Row, Glasgow, G1 1XJ, U.K.*
**ralf.bauer@strath.ac.uk*

MEMS-SIM 3D schematic and parts list

The supplemental material of the presented Microelectromechanical System (MEMS) structured illumination microscopy (SIM) system contains a detailed 3D schematic of the system in Fig. S1, as well as a complete parts list in Table S2.

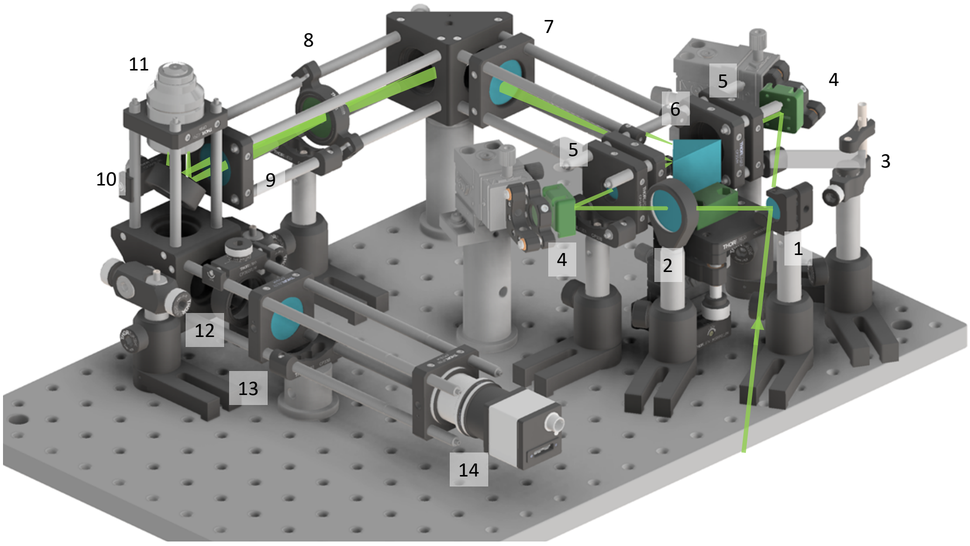

Fig. S1. 3D schematic of the MEMS-SIM system, with illumination beam in green. The sample holder and stage are left out for clarity. 1: 50/50 beam splitter, 2: compensation plate, 3: continuous variable ND filter, 4: MEMS mirrors, 5: f = 30 mm ½" diameter achromatic lenses, 6: knife edge prism, 7: f = 125 mm 1" diameter achromatic lens, 8: aperture, 9: f = 75 mm 1" diameter achromatic lens, 10: multi-band dichroic beam splitter, 11: 100x 1.25NA Zeiss objective, 12: multi-band emission filter, 13: f = 175 mm achromatic tube lens, 14: IDS UI-3060CP industrial CMOS

Table S2. Parts and costs for assembling a full MEMS-SIM system including two lasers

#	Supplier	Item	Description	No	Item price (£)	Overall price (£)
1	Odicforce	OFL17-F2	150mW 532nm laser module	1	40.83	40.83
2	Lasever Inc	LSR473NL-50-PS-II	50mW 473nm diode pumped solid state laser	1	490.83	490.83
3	Mirrorcle	A7M20.2-2000AL	2mm MEMS mirror	2	429.17	858.33
4	Edmund Optics	#13-821	100x 1.25NA Zeiss A-Plan Oil Immersion Objective	1	593.00	593.00
5	IDS	UI-3060-CP-M-GL Rev.2	IDS CMOS camera 1936x1216px	1	480.00	480.00
6	Chroma	DC/ZT375/473/532/635rpc-UF1	Dichroic filter (multiband)	1	654.00	654.00
7	Chroma	69401m	Emission filter (multiband)	1	347.00	347.00
8	Thorlabs	MRAK25-P01	Knife edge prism	1	102.32	102.32
9	Thorlabs	BSW04	50/50 1/2" broadband beam splitter	1	64.26	64.26
10	Thorlabs	NDL-10C-2	Continuous variable ND filter	1	65.51	65.51
11	Thorlabs	BCP4310	Beamsplitter compensation plate, 5mm thick	1	80.69	80.69
12	Thorlabs	AC127-030-A	f=30mm 1/2" lenses	2	42.43	84.86
13	Thorlabs	AC254-125-A	f=125mm 1" lens	1	62.39	62.39
14	Thorlabs	AC254-075-A	f=75mm 1" lens	1	62.39	62.39
15	Edmund Optics	#47-644	f=175mm 1" tube lens	1	90.10	90.10
16	Thorlabs	ME1-P01	1" Ag mirror	2	24.00	48.00
17	Thorlabs	ME05-P01	1/2" Ag mirror	3	13.13	39.39
18	Thorlabs	DMLP505T	1/2" dichroic laser beam combiner	1	94.01	94.01
19	Thorlabs	LPVISE2X2	Economy polariser	1	6.82	6.82
20	Thorlabs	KPZNFL5/M	Piezo z-stage	1	1182.93	1182.93
21	Thorlabs	MT1A/M	Manual xy-stage	2	326.11	652.22
22	Thorlabs	MT401/M	xy base plate	1	18.83	18.83
23	Thorlabs	NFL5P2/M	z-stage angle bracket	1	80.69	80.69
24	Thorlabs	DT12XYZ/M	MEMS 3-axis position stage	2	252.06	504.12
25	Thorlabs	DT12XY/M	Knife edge prism 2-axis positioning stage	1	153.89	153.89
26	Thorlabs	KM100B/M	Tip-Tilt platform for knife edge prism	1	46.39	46.39
27	Thorlabs	MSRP01/M	Rotation mount for knife edge prism	1	59.69	59.69
28	Thorlabs	RSP1/M	Rotation mount for linear polariser	1	70.30	70.30
29	Thorlabs	KCB1C/M	Right angle cage mirror mounts	2	110.47	220.94
30	Thorlabs	CP360R/M	360deg pivot cage mirror mount	1	78.00	78.00
31	Thorlabs	CP35/M	1" optics cage plate	5	15.08	75.40
32	Thorlabs	CP33/M	1" threaded optics cage plate	1	13.64	13.64
33	Thorlabs	CP36	1.2" optics cage plate	1	16.97	16.97
34	Thorlabs	CP33B	Cage mounting bracket	4	11.48	45.92
35	Thorlabs	CMT10	SM1 thread to C-mount adapter	1	16.97	16.97
36	Thorlabs	ER4-P4	4" long cage rods	4	20.84	83.36
37	Thorlabs	ER3-P4	3" long cage rods	1	19.37	19.37

38	Thorlabs	ER2-P4	2" long cage rods	1	17.91	17.91
39	Thorlabs	ER1-P4	1" long cage rods	1	14.83	14.83
40	Thorlabs	KM05/M	1/2" 2-axis mirror holder	6	30.13	180.78
41	Newport	U50-S	1/2" 2-axis mirror holder clear edge	1	63.00	63.00
42	Thorlabs	RS2.5P/M	2.5" long 1" pedastal post	2	23.50	47.00
43	Thorlabs	RS1P/M	1" long 1" pedastal post	1	18.72	18.72
44	Thorlabs	UPH40/M-P5	Universal 1/2" post holder 5pack	3	122.70	368.10
45	Thorlabs	RA90/M	90deg 1/2" post clamps	3	7.73	23.19
46	Thorlabs	TR50/M-P5	50mm long 1/2" post 5pack	4	18.95	75.80
47	Thorlabs	TR20/M-P5	20mm long 1/2" post 5pack	1	17.30	17.30
48	Thorlabs	MB2530/M	Breadboard	1	109.81	109.81
49	Misc	3D-prints	3D-printed adapters for MEMS, stage, pinholes, etc	1	16.14	16.14
50	RS	Arduinio Micro	Arduino Micro microcontroller	1	17.88	17.88
51	Digikey	EVAL-AD5676RSDZ	8-channel DAC evaluation board	1	91.77	91.77
52	RS	HV56264	225V amplifier HV56264	2	60.63	121.26
53	RS	AC/DC adapter	24V AC/DC adapter	1	9.50	9.50
54	RS	AD8694ARZ	AD8694ARZ 5th order low pass filter	2	2.57	5.14
55	RS	Misc	Voltage regulators, resistors, capacitors	3	5.00	15.00
					Total:	£ 8817.49

Full images of varying MEMS-SIM grating periods for bead and cell samples

Fig. S3 shows the full field images of the green 175 nm nanobead samples (PS-Speck Point Source Kit P7220, Invitrogen) excited with the 473 nm laser with (A) showing the SIM image using a grating period of 660 nm, (B) a SIM image with grating period 550 nm and (C) a SIM image with grating period of 440 nm. Fig. S3 (D) and (E) show the summed widefield image and a Wiener filtered widefield image of the same bead area. Fig. S4 shows the full field images of the orange 175 nm nanobead samples (PS-Speck Point Source Kit P7220, Invitrogen) excited with the 532 nm laser, again with (A) showing the SIM image with a grating period of 660 nm, (B) showing the SIM image with a grating period of 550 nm and (C) with a grating period of 440 nm. The second row shows also again in (D) the summed widefield image and in (E) the Wiener filtered widefield image of the same area.

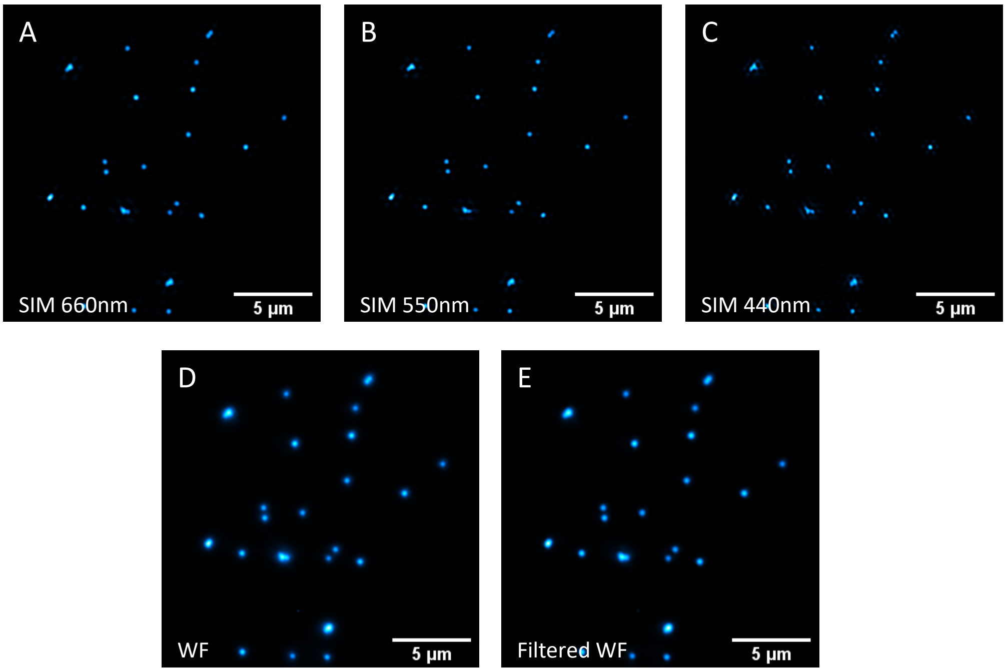

Fig. S3. 175 nm green fluorescence beads excited with 473 nm laser and recovered with different SIM grating periods. (A) 660 nm grating period, (B) 550 nm grating period, (C) 440 nm grating period, (D) summed widefield image, (E) Wiener filtered widefield image.

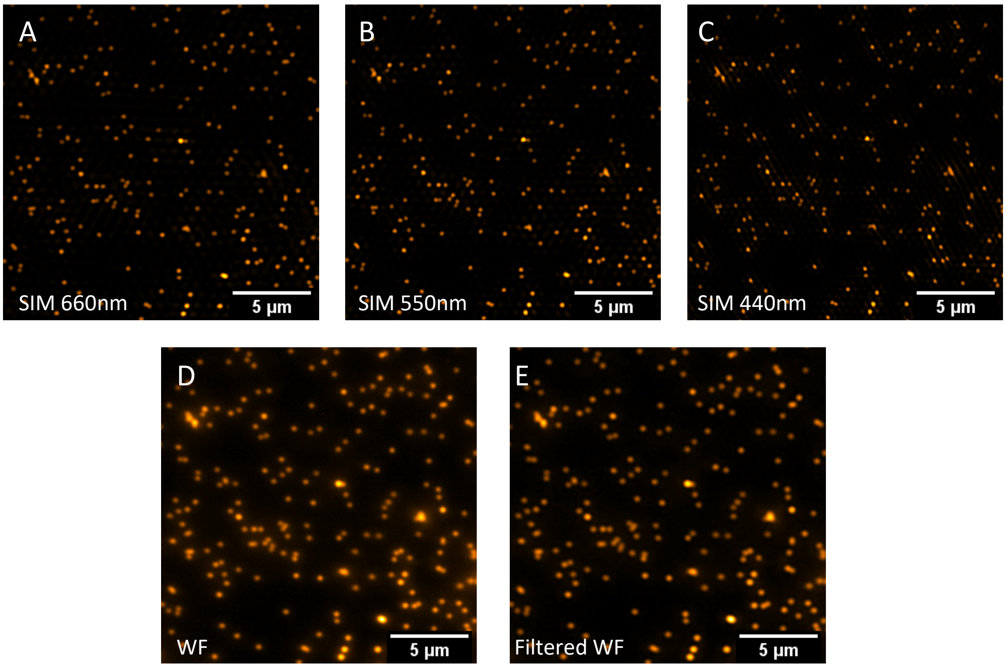

Fig. S4. 175 nm orange fluorescence beads excited with 532 nm laser and recovered with different SIM grating periods. (A) 660 nm grating period, (B) 550 nm grating period, (C) 440 nm grating period, (D) summed widefield image, (E) Wiener filtered widefield image.

Fig. S5 shows an example are of a fixed BPAE cell slide image for both the 473 nm excitation (exciting Alexa Fluor 488 phalloidin labelled F-actin in blue) and the 532 nm excitation (exciting Mitotracker Red CMXRos labelled mitochondria in orange), ranging from the reconstructed widefield image over the 660 nm grating, 550 nm grating to the 44 0nm grating image reconstructed with fairSIM.

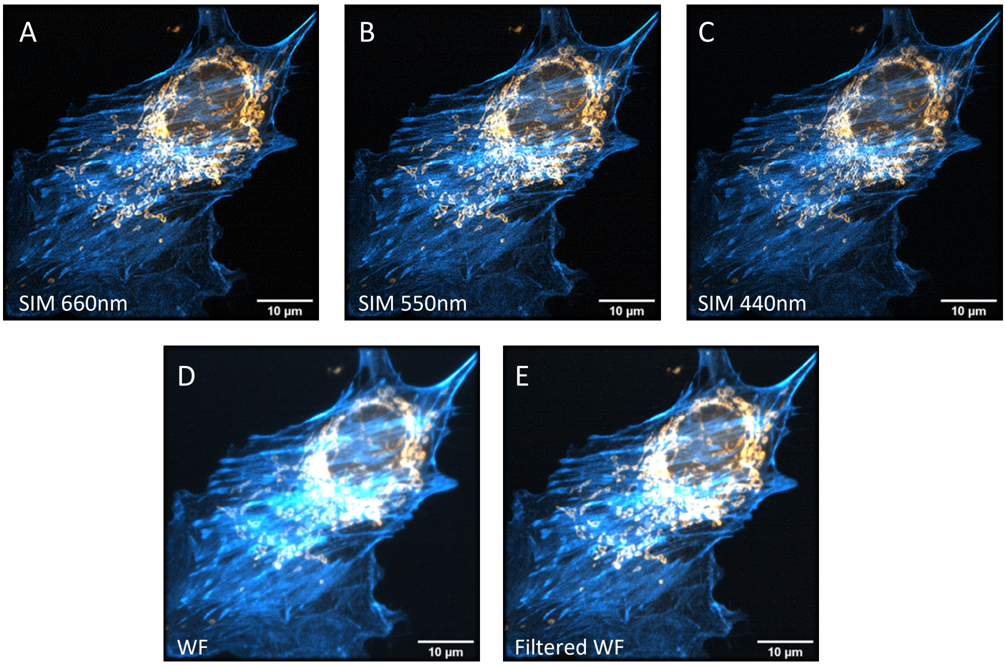

Fig. S5. Dual color SIM reconstruction and summed widefield image of a fixed BPAE cell slide with blue showing the actin network and orange showing mitochondria. (A) SIM reconstruction using 660 nm grating period for both excitation wavelengths, (B) SIM reconstruction using a 550 nm grating period, (C) SIM reconstruction using a 440 nm grating period, (D) summed widefield image of the same are and (E) Wiener filtered summed widefield.

MEMS control electronics

To control the position and movement of the two MEMS mirrors in their three axes (tip, tilt and piston) a custom electronics schematic is used, based on commercially available evaluation boards and simple signal filtering circuits. The overview schematic of the electronics is shown in Fig. S6.

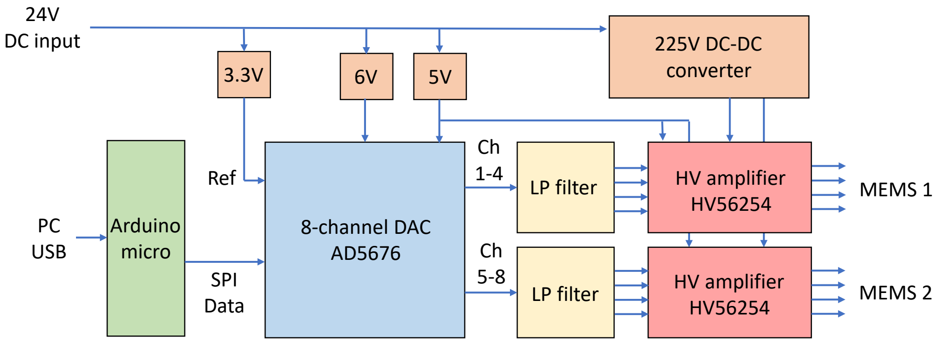

Fig. S6. Electronics schematic.

The electronics consist of an Arduino micro connected to a control PC via a USB serial connection, a 8-channel DAC board (EVAL-AD5676, Analog Devices) for generation of the driving signals of the four actuators of each MEMS, two high voltage amplifiers (HV56264, Microchip) to generate the up to 180 V drive voltages of the MEMS, a 24 V AC/DC adapter as power supply (175-3312, RS), a 5 V voltage regulator (MC78M05CTG, Onsemi) to power the

DAC and amplifier control inputs, a 6 V voltage regulator (NJM7806DL1A-TE1, Nisshinbo Micro Devices) as second input voltage to the DAC, a 3.3 V voltage regulator (AP2114D-3.3TRG1, DiodesZetex) to provide a reference voltage for the DAC levels, a 225 V DC/DC boost converter (ZVS step-up boost converter 45-390 V, Ebay) to power the high voltage amplifiers and a passive component 2nd order low-pass filter (using a 10 kΩ resistor and 47 nF capacitor in the first stage and a 20 kΩ and 33 nF capacitor in the second stage) for each DAC output. The Arduino controls the 8 output channels of the DAC using a single SPI connection, with each output having their own low pass filter and four DAC outputs being amplified by one of the 4-channel high voltage amplifiers each.

Back focal plane images of SIM grating excitation beams

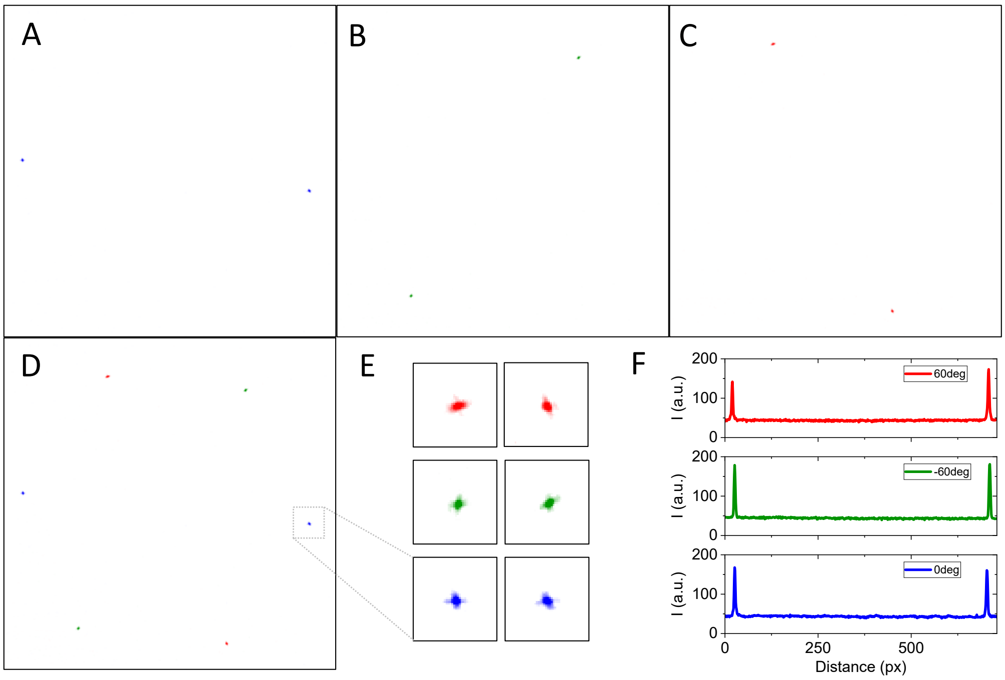

Fig. S7. Images of the 440 nm grating spacing excitation laser pattern near the objective back focal plane. (A) Pattern for 0° orientation; (B) Pattern for -60° orientation; (C) Pattern for 60° orientation; (D) Composite of all three grating orientations; (E) Zoom-in on the spot sizes of each grating orientation; (F) Cross-section through all three directions.
